# Schottky solar cell using few-layered transition metal dichalcogenides toward large-scale fabrication of semitransparent and flexible power generator

**DOI:** 10.1038/s41598-017-12287-6

**Published:** 2017-09-20

**Authors:** Toshiki Akama, Wakana Okita, Reito Nagai, Chao Li, Toshiro Kaneko, Toshiaki Kato

**Affiliations:** Department of Electronic Engineering, Tohoku University, Aoba 6-6-05, Aramaki, Aoba-ku, Sendai 980-8579 Japan

## Abstract

Few-layered transition metal dichalcogenides (TMDs) are known as true two-dimensional materials, with excellent semiconducting properties and strong light–matter interaction. Thus, TMDs are attractive materials for semitransparent and flexible solar cells for use in various applications. Hoewver, despite the recent progress, the development of a scalable method to fabricate semitransparent and flexible solar cells with mono- or few-layered TMDs remains a crucial challenge. Here, we show easy and scalable fabrication of a few-layered TMD solar cell using a Schottky-type configuration to obtain a power conversion efficiency (PCE) of approximately 0.7%, which is the highest value reported with few-layered TMDs. Clear power generation was also observed for a device fabricated on a large SiO_2_ and flexible substrate, demonstrating that our method has high potential for scalable production. In addition, systematic investigation revealed that the PCE and external quantum efficiency (EQE) strongly depended on the type of photogenerated excitons (A, B, and C) because of different carrier dynamics. Because high solar cell performance along with excellent scalability can be achieved through the proposed process, our fabrication method will contribute to accelerating the industrial use of TMDs as semitransparent and flexible solar cells.

## Introduction

Transparent or semitransparent solar cells with excellent mechanical flexibility have attracted much attention as next-generation smart solar cells for various applications such as in the surfaces of windows, front display panels of personal computers and cell phones, and human skin^1–3^. However, despite recent progress in the fabrication of solar cells^4,5^, critical issues remain with regard to their practical applications, such as improving their power conversion efficiency (PCE), optical transparency, flexibility, stability, and scalability. Because most of these issues involve materials, the development of new photovoltaic materials with high transparency and mechanical flexibility is required.

Transition metal dichalcogenides (TMDs) are known as true two-dimensional (2D) materials with excellent semiconducting properties and strong mechanical flexibility, in addition to being atomically thin^6–8^. TMDs with thicknesses of less than 1 nm show strong light–matter interactions, affording absorption of incident sunlight of as much as 5–10%, which is one order of magnitude higher than that of common semiconductors such as GaAs and Si^9^. These features make TMDs among the most attractive materials for high-performance, semitransparent, and flexible solar cells.

Fabrication of solar cells using TMDs has been investigated by many groups^10–22^. The fabrication can be divided into two types: in one type, TMDs are used as PCE enhancers, wherein the TMDs are combined with other solar cell materials, such as Si and GaAs, where a relatively high PCE can be obtained even without TMD^10–12^. In the other type of fabrication, only TMDs are used as the photoactive layers. Because the first type (hetero-junctions of TMDs with other materials) deteriorates the original high transparency and mechanical flexibility of TMDs, the second type (using TMD only) is necessary for the application of semitransparent and flexible solar cells. Table S1 in the Supplementary Data summarizes the results of the use of TMDs as photoactive layers. A high PCE of 14% and a high external quantum efficiency (EQE) of 75% can be obtained by using thick TMD crystals; however, thick crystals are not optically transparent^16,17^. For thinner TMDs, those with less than three layers (L), the PCE is limited, showing very low values (~0.01%) for a pn-junction solar cell containing a single layer of WSe_2_^13,15^ and solar cells with a vertically stacked heterojunction (WSe_2_/MoS_2_)^19,20^. Although a PCE of 0.5% was reported, the PCE was not measured by a standard method (light source and irradiation power)^14^. The use of pn- or hetero-junction solar cells requires the use of complicated device structures and fabrication processes, which restricts the device size to the micrometer scale. Thus, developing a scalable method with mono- or few-layered TMDs on a transparent, flexible substrate is required to realize semitransparent and flexible solar cells with true 2D materials.

The Schottky-type solar cell has a simple structure, and thus it is possible to scale up the device to the industrial wafer scale. Despite having this technical advantage, a detailed study of solar cells containing mono- or few-layered TMDs has not yet been carried out; only one study on solar cells containing thick MoS_2_ (50 nm) has been reported^22^.

Because a Schottky barrier is formed at the contact region between an electrode and a TMD, it is important to use appropriate electrode pairs for the left and right electrodes (asymmetric electrodes). In this study, we investigated in detail the effects of various device structures (combination of left and right electrodes and the distance between each electrode) and TMD morphologies (“on substrate” or “suspended”) on the photovoltaic features of the solar cell to obtain a better device performance. By varying these factors, we achieved the fabrication of Schottky-type solar cells with a few-layered TMD. The solar cells fabricated on a SiO_2_ substrate demonstrated a high PCE of ∼0.7%, which is the highest for solar cells with thin (below 3 L) TMDs^13–15,19–21^. In addition, the scalability of our method is also demonstrated through the fabrication of solar cells with few-layered WS_2_ on a centimeter-scale SiO_2_ and flexible substrate, where clear power generation is demonstrated. The photovoltaic mechanism was investigated in detail using EQE spectroscopy, differential reflectance spectroscopy, scanning Kelvin probe force microscopy (SKPM), and photocurrent mapping with multi-wavelength excitation. The photocurrent map showed that the effective area used for photocurrent generation strongly depended on the incident photon energy, which can be explained by the difference in exciton diffusion dynamics of the A, B, and C excitons in TMD.

## Results and Discussion

### Fabrication of a high-performance Schottky solar cell with a few-layered TMD

**Figure 1:**
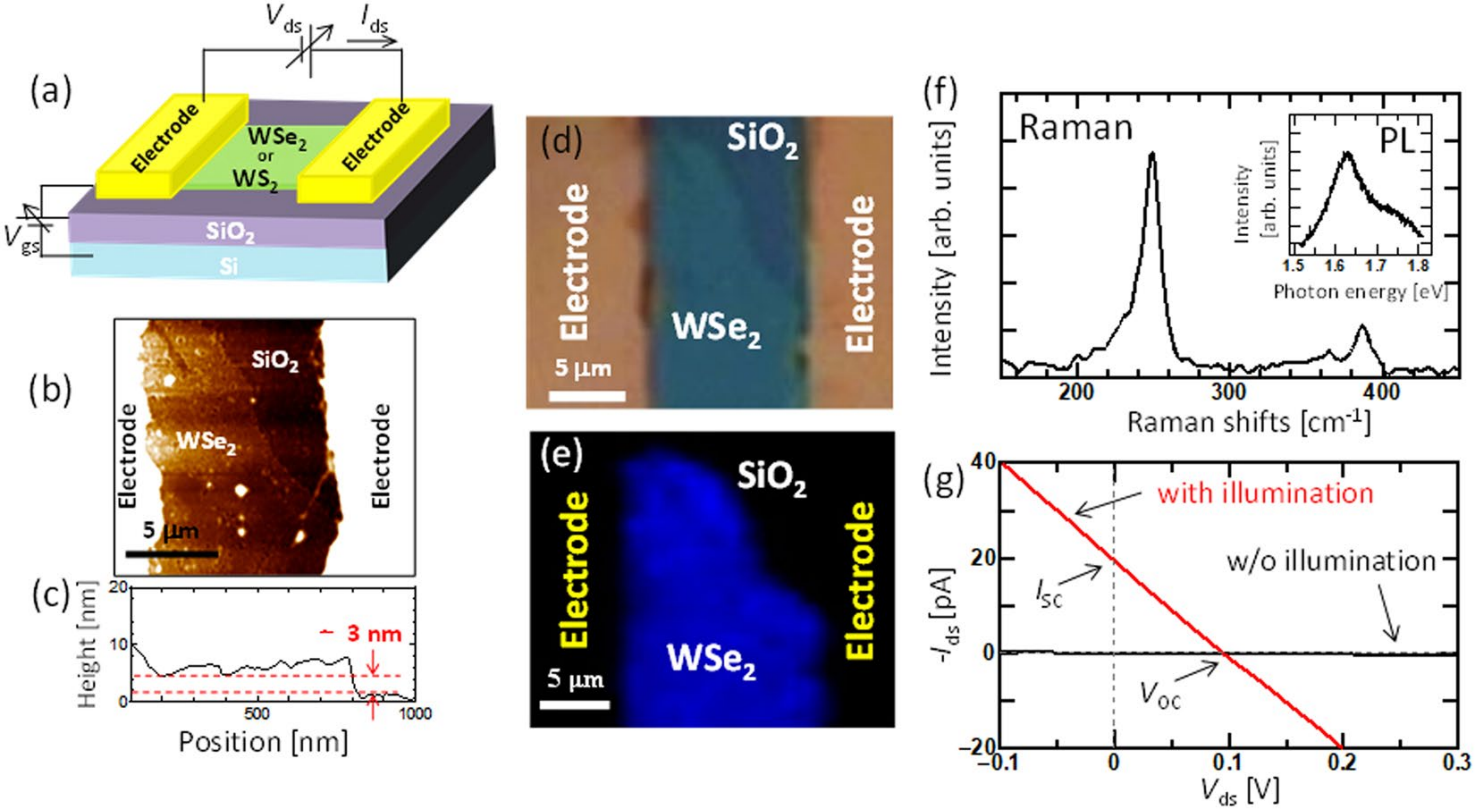
Solar cell of few-layer WSe_2_ with a symmetric electrode. (**a**) Schematic illustration of the device structure. (**b**–**g**) Typical (**b**) AFM image, (**c**) height profile, (**d**) optical microscope image, (**e**) Raman intensity mapping image, (**f**) Raman scattering spectra, and (inset in **f**) PL spectra of few-layered WSe_2_. (**g**) *I*_ds_ − *V*_ds_ characteristics of few-layered WSe_2_ with (red) and without (black) right (solar simulator) illumination.

**Figure 2:**
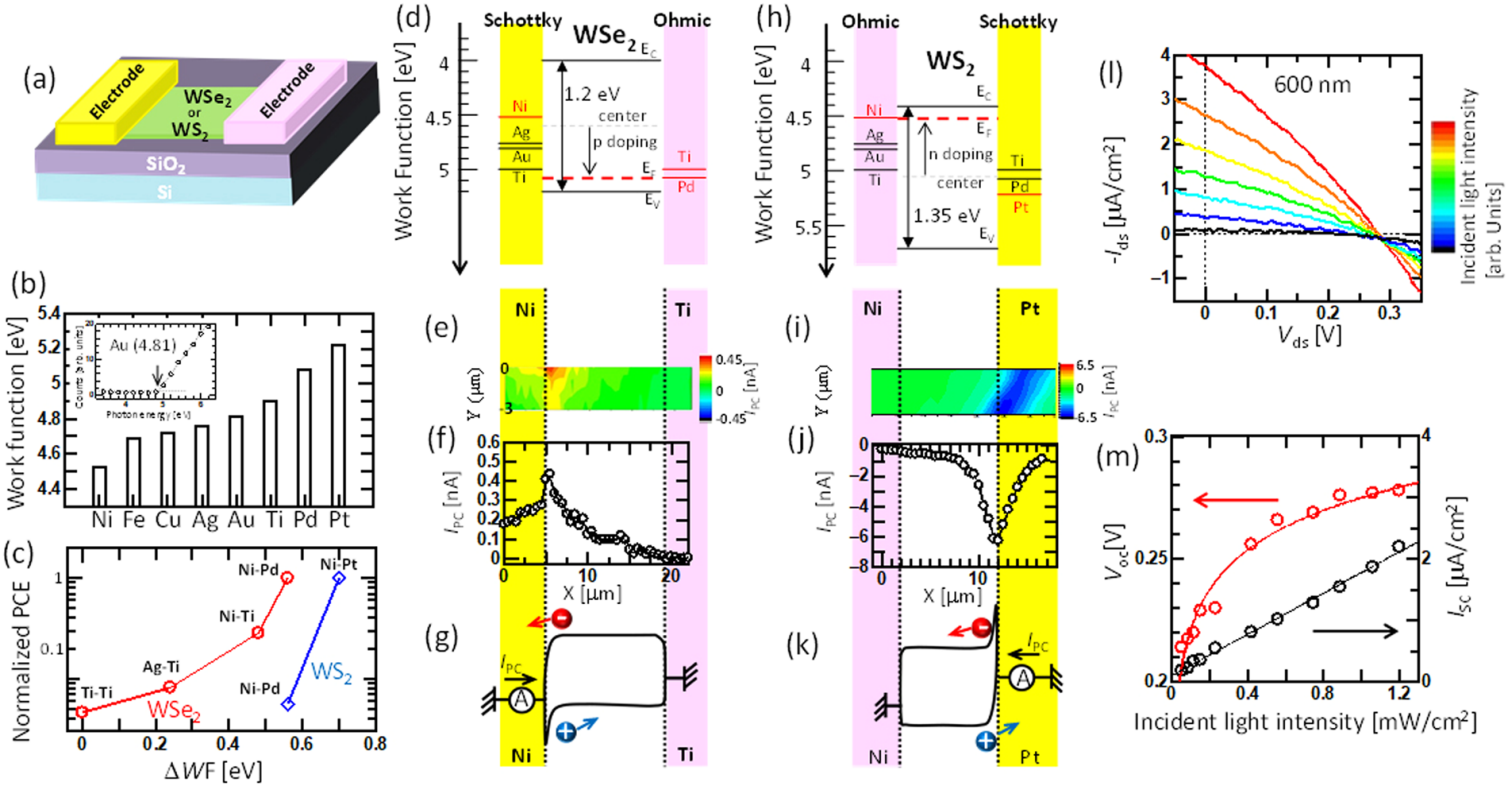
Solar cell of few-layer TMD (WSe_2_ or WS_2_) with an asymmetric electrode. (**a**) Schematic illustration of the device structure. (**b**) Plot of the work function for various metals measured by photoelectron yield spectroscopy. Inset in (**b**) shows the typical photoemission features of Au film and the fitting curve to obtain the work function. (**c**) Normalized PCE as a function of ΔWF between the asymmetric electrode pairs for WSe_2_ (red) and WS_2_ (blue). (**d**–**k**) (**d**,**h**) Band diagram, (**e**,**i**) photocurrent mapping, (**f**,**j**) photocurrent line scan, (**g**,**k**) band structure of few-layered (**d**–**g**) WSe_2_ and (**h**–**k**) WS_2_ asymmetric devices. (**l**,**m**) (**l**) Typical *I*_ds_ − *V*_ds_ features and (**m**) *I*_sc_ (red) and *V*_oc_ (black) of few-layered WSe_2_ with asymmetric electrode pairs under different incident light (600 nm) intensities.

A Schottky-type solar cell was fabricated via mechanical exfoliation from bulk TMD crystals (WSe_2_ and WS_2_) and chemical vapor deposition (CVD)-grown WS_2_ combined with conventional photolithography and electron-beam lithography (see the Methods section for more detailed information). The photovoltaic features were measured with symmetric Ti electrodes (Ti electrode was covered by 30 nm Au to protect Ti surface from oxidation) (Fig. 1a). In this study, bi-layered and tri-layered TMDs (WSe_2_ and WS_2_) were used. The data shown in this manuscript correspond to tri-layered WSe_2_ and WS_2_ unless otherwise specified. The structures of WSe_2_ and WS_2_ were characterized by atomic force microscopy (AFM) (Fig. 1b,c), optical microscopy (OM) (Fig. 1d), Raman scattering (Fig. 1e,f), and photoluminescence (PL) spectroscopy (inset in Fig. 1f). The three terminal device configuration with source, drain, and gate electrodes was used in this study. Gate bias voltage (*V*_gs_) was applyded from the back side of highly-doped Si substrate through the insulating layer (SiO_2_: 300 nm) and additional air gap (a few tends nm; only for suspended device). Although using gate bias is not practical for industrial applications of transparent solar cells, the gate bias can be replaced by chemical doping, which can be one of the future subjects in this study. The source–drain current (*I*_ds_) vs. source–drain voltage (*V*_ds_) curve with light illumination was measured for the symmetric source and drain electrode (Ti). A clear short-circuit current (*I*_sc_) and open-circuit voltage (*V*_oc_) were observed (Fig. 1g). Based on the photocurrent mapping measurements (Fig. S1), we confirmed that power generation occurred only at the contact region between the electrode and WSe_2_, indicating that Schottky-type power generation occurred in this device (as discussed below). Because the photogenerated carriers travel in the opposite direction at both ends of the electrode, the photocurrent generated at the left and right sides of the electrode should, in principle, cancel out, resulting in zero power generation. Therefore, the power generation obtained must be caused by inhomogeneous contact between the left and right electrodes. By following this model, an ideal structure can be realized, where only one of the electrodes generates carriers with the Schottky barrier and the other electrode effectively collects the carriers with an ohmic-like contact. Then, we attempted to find a suitable electrode pair for Schottky-type solar cells with WSe_2_ and WS_2_ (Fig. 2a). Because the contact structure between the electrode and WSe_2_ (WS_2_) is basically governed by the work function difference of each material, we systematically measured the work function (WF) of various metals used as electrodes by photoelectron yield spectroscopy. Figure 2b shows the obtained WF values for various metals. Since “as exfoliated few-layered WSe_2_ (WS_2_)” is naturally p-doped (n-doped) (Fig. S2) by some impurities, the Fermi energy of our WSe_2_ (WS_2_) can be assumed to be approximately 5 (4.5) eV^23,24^. Thus, Ti (WF = 4.9 eV) or Pd (WF = 5.08 eV) can work as an ohmic contact for few-layered WSe_2_, whereas Ni (WF = 4.52 eV), which has the lowest work function in this measurement, can form a large Schottky barrier at the contact region with WSe_2_ (Fig. 2d,h). Because the accurate electrical band structure of few-layered TMD is still unclear, we used the optical band of the few-layered TMD for the discussion of the band diagram instead of the electrical band. The similar correlation (Schottky or ohmic-like) between each electrode and TMD can be also obtained by considering the electrical band of monolayer TMD (Fig. S3). A Schottky-type solar cell with an asymmetric electrode pair was fabricated with various electrodes. A clear difference in *I*_ds_ − *V*_ds_ was observed depending on the electrode pair under light illumination with a solar simulator. The normalized PCE was plotted as a function of the WF difference between the left and right sides of the electrode (ΔWF) (Fig. 2c). For PCE calculation, we used the area of TMD bridging between the two electrodes as an active area. The detailed method for PCE calculation is shown in Fig. S4. The efficiency clearly depended on ΔWF, and a higher efficiency could be obtained with higher ΔWF (Pd–Ni), which is consistent with our concept, where Ni and Pd can form large and small Schottky barriers to operate as power-generation and carrier-collection regions, respectively. Note that it is reported that Fermi level pinning can also decide the contact barrier between electrode and TMD, causing weak WF dependence (pinning factor: *S* = 0.1−0.3)^25–27^. Because the defects on the surface of TMD can be considered as the critical cause for Fermi level pinning at TMD surface, decreasing the defect density in TMD can contribute to the improvement of *S*, resulting in higher device performance for a Schottky-type solar cell.

To support the accuracy of these explanations, we attempted to identify the band structure of the asymmetric electrode contacts via a photocurrent mapping measurement^28–30^. First, the photocurrent mapping measurement was carried out with symmetric electrodes (Ti-Ti) (Fig. S1). The Raman and PL spectra were measured at the same time with photocurrent measurements, which enabled us to identify the power generation region by comparison with the Raman and PL mapping results^30^. Positive and negative currents were generated only near the left and right electrode regions, respectively. The *I*_ds_ − *V*_gs_ curve for this device showed typical p-type transport properties (Fig. S2). Thus, the Schottky barrier was formed for the holes near the contact region (Fig. S1e). Then, similar measurements were carried out for the asymmetric electrode (Ni-Ti). The photocurrent was observed only near the Ni electrode region (Fig. 2e,f), which demonstrates that the Schottky barrier only formed in the Ni-WSe_2_ contact region (Fig. 2g). To confirm the accuracy of the band structure for the asymmetric electrode, photocurrent mapping measurements were also performed for the symmetric electrode (Ti-Ti) with *V*_ds_ = 1 V. In this case, a photocurrent was obtained only near the electrode where positive *V*_ds_ was applied (Fig. S5). It is known that the band structure in this case becomes that shown in Fig. S5d ^28^. Because the photocurrent features of the asymmetric electrode (Fig. 2e,f) are similar to those of the symmetric electrode with positive bias (Fig. S5b,c), the band structure shown in Fig. 2g is reasonable. A similar experiment was carried out for WS_2_. The highest PCE was obtained with the Ni–Pt electrode pair for WS_2_ (Fig. 2c). The photocurrent mapping revealed that a photocurrent could be generated only around the contact region between Pt and WS_2_ (Fig. 2i,j). The *I*_ds_–*V*_gs_ curves of the WS_2_ device showed n-type features (Fig. S2). These results indicated that a Schottky barrier formed only at the conduction band of the Pt side, whereas an ohmic-like contact formed on the Ni side (Fig. 2k). This is also consistent with the band diagram shown in Fig. 2h. The data for WS_2_ also support the accuracy of our concept of asymmetric contact to obtain better device performance with a Schottky-type solar cell.

The dependence of the incident light intensity on the sample was also measured with asymmetric electrodes for WSe_2_. The *I*_ds_ − *V*_ds_ curves clearly changed with increasing light intensity (Fig. 2l). *I*_sc_ showed a linear correlation with light intensity, whereas *V*_oc_ showed saturated features, which are typical for photovoltaic power generation (Fig. 2m). This shows that photothermal power generation could be negligible in our device^13^. Similar results were obtained with the Ni–Pt asymmetric electrodes for WS_2_ (Fig. S6).

**Figure 3:**
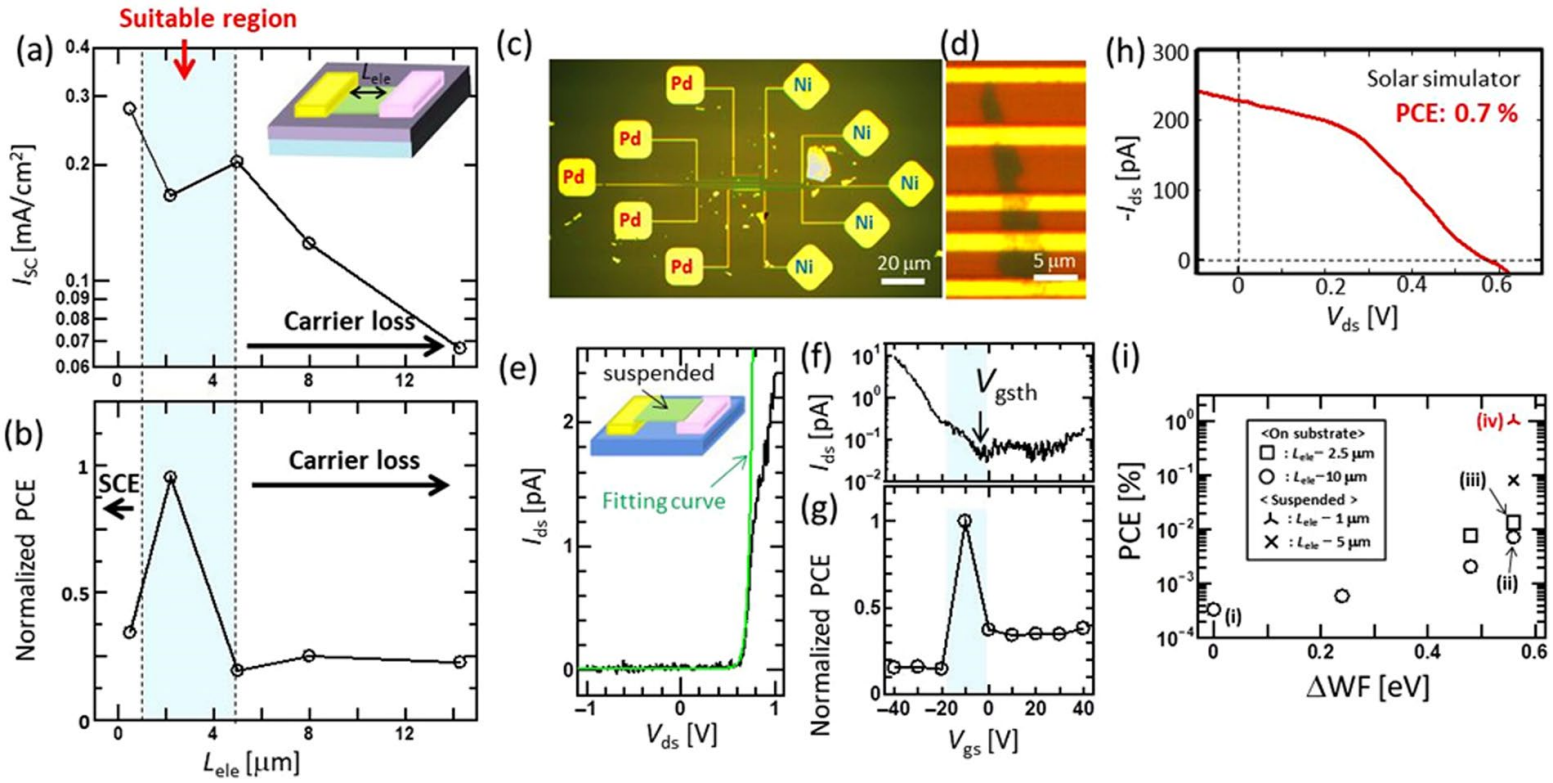
Photovoltaic features of a high-performance few-layered WSe_2_ solar cell. (**a**,**b**) (**a**) *I*_sc_ and (**b**) normalized PCE for different *L*_ele_. *I*_sc_ in (**a**) was normalized by each sample area. (**c**–**e**) Typical (**c**) low- and (**d**) high-magnification optical microscope images and (**e**) *I*_ds_ − *V*_ds_ curves without light irradiation of a suspended WSe_2_ device with asymmetric electrodes. Green curve in (**e**) shows the fitting curve for the diode equation. (**f**,**g**) (**f**) *I*_ds_ and **(g**) normalized PCE of a suspended TMD device as a function of *V*_gs_. (**h**) Typical *I*_ds_ − *V*_ds_ curves of a suspended TMD device under light irradiation with a solar simulator. (**i**) Plots of PCE vs. ΔWF of various TMD solar cell devices, where (i)–(iv) denotes (i) a symmetric electrode (Ti-Ti) with long *L*_ele_, (ii) a Pd-Ni electrode with long *L*_ele_, (iii) a Pd-Ni electrode with short *L*_ele_, and (iv) a Pd-Ni electrode with suspended TMD, respectively.

Next, we investigated the effects of the electrode distance between the left and right electrodes (*L*_ele_). Various asymmetric electrode devices were fabricated with Ni–Ti asymmetric electrodes by changing *L*_ele_. *I*_sc_ monotonically decreased with increasing *L*_ele_ over 5 μm. However, a significant variation in *I*_sc_ was not observed for relatively short *L*_ele_ values (0.5 to 5 μm) (Fig. 3a). This can be explained by the longer *L*_ele_ causing carrier loss due to the recombination of excitons or photogenerated carriers (see below for a more detailed mechanism). The power generation efficiency also showed a similar trend, where a longer *L*_ele_ led to a low efficiency (Fig. 3b). The shortest *L*_ele_ (0.5 μm) showed a lower efficiency even with the highest *I*_sc_ value, which may be explained by the low *V*_oc_. Because the value of *V*_oc_ strongly correlates with shunt resistance, the decrease of the shunt resistance may be one of the possible reasons for this lower *V*_oc_. The critical reason for the decrease of shunt resistance with short channel device (*L*_ele_ = 0.5 μm) is not sure at the current state. Through systematic investigations, we found that a *L*_ele_ value of apploximately 1–2 μm led to the highest PCE.

We also measured the effect of the substrate on the photovoltaic features of few-layered WSe_2_. A suspended WSe_2_ layer with an asymmetric electrode (Pd–Ni) was fabricated (Fig. 3c,d) (see the Methods section and Fig. S7 for more details). Clear rectification features were observed, which were well fitted by the Shockley diode equation^14^, ${\rm{I}}={I}_{s}(\exp (V/n{V}_{T})-1)$, where *I*_*S*_, *V*, n, and *V*_*T*_ denote the saturation current, the voltage between the left and right electrodes, the diode ideality factor (*n* = 1 is ideal), and the thermal voltage at temperature *T*, respectively (Fig. 3e). The fitting gave *n* = 1.5, indicating that the suspended few-layered WSe_2_ on an asymmetric electrode showed excellent diode features. The PCE showed the highest value at a threshold voltage of *V*_gs_ (*V*_gsth_ = −10 V) for *I*_ds_ (Fig. 3f,g) because the Schottky barrier height could be modulated by *V*_gs_, and the highest PCE was achieved when the Schottky height reached a maximum near the *V*_gsth_ condition. A typical *I*_ds_–*V*_ds_ curve under light illumination showed a relatively high *V*_oc_ = 0.58 V (Fig. 3h). The PCE under the adjusted device configuration could be increased up to 0.7% with solar simulator irradiation, indicating that the suppression of substrate effects such as carrier scattering caused by substrate impurities can drastically improve the solar cell performance, which is consistent with pn- and heterojunction devices with h-BN as an interlayer^13,16^. Because the *I*_ds_ − *V*_ds_ curve showed almost the same features with forward and reverse bias sweep (Fig. S8), the charging effect at the surface or interlayer of the few-layered WSe_2_ device is considered negligible.

Figure 3i shows a summary of normalized PCE values for various electrode structures (detailed values are shown in Table S2.). The PCE of all devices was measured by changing V_gs_ from −40 to 40 V. Then, we used the maximum value for each device within different V_gs_ values for the comparison of PCE. A higher ΔWF led to a higher PCE, as already shown in Fig. 2c. The suspended TMD on an asymmetric electrode gave a PCE value that was more than 3000 times higher than that before the adjustment of the electrode structures. The PCE reached 0.7% (active area: 7 μm^2^) measured with a solar simulator. To the best of our knowledge, this efficiency (0.7%) is the highest value for TMD-based solar cells with similar thicknesses (Table S1). Because the Schottky-type solar cell has a simple structure, it is easy to scale the device up to practical sizes. We believe that our findings are very important to realizing the practical application of semitransparent and flexible solar cells with TMDs.

### Large-scale fabrication of a semitransparent solar cell with few-layered WS_2_

**Figure 4:**
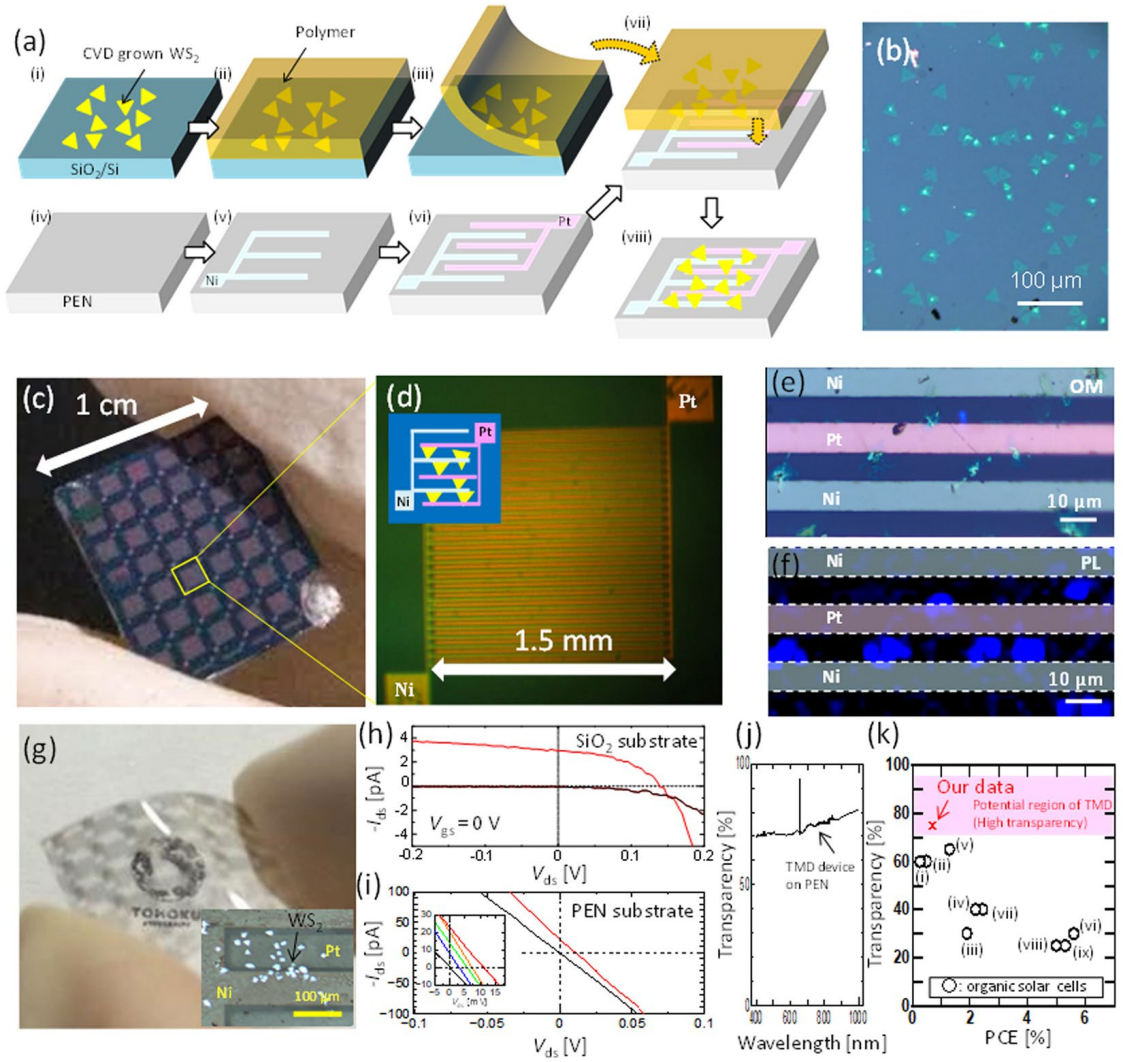
Large-scale fabrication of a Schottky-type solar cell with few-layered WS_2_. (**a**,**b**) (**a**) Typical fabrication process of a large-scale solar cell with our method and (**b**) optical microscope image of CVD-grown WS_2_ used in this study. (**c**,**d**) (**c**) Low- and (**d**) high-magnification images of a large-scale Schottky type solar cell on a SiO_2_ substrate. Multiple solar cell units with asymmetric electrode pairs (Ni-Pt) were patterned over the entire substrate region. **(e**,**f**) (**e**) High-magnification optical microscope image and (**f**) PL intensity mapping of specific channel and electrode regions in an asymmetric electrode device. (**g**) Optical image of a Schottky-type solar cell with few-layered WS_2_ fabricated on a transparent and flexible substrate. Inset in (**g**) shows the high magnification OM image of WS_2_ briding between Pt and Ni electrode on a PEN substrate. (**h**,**i**) Typical *I*_ds_ − *V*_ds_ features with and without light (solar simulator) illumination for a specific asymmetric device fabricated on a large (**h**) SiO_2_ substrate and (**i**) PEN substrate. Inset in (**i**) denotes *I*_ds_ − *V*_ds_ curve of the PEN device measured by different power of solar simulator (black: 0, blue: 0.05 W/cm^2^, green: 0.1 W/cm^2^, orange: 0.14 W/cm^2^, red: 0.2 W/cm^2^). (**j**) Transparent spectra of a WS_2_-based solar cell with an asymmetric electrode on a PEN substrate. (**k**) Comparison of transparency vs. PCE with our TMD-based device and other organic solar cells. Data of ○ (i)–(ix) are replotted using the data from ref.^1,2,4,31–35^ respectively.

Because a relatively high-performance solar cell could be fabricated with few-layered WSe_2_ and WS_2_ by a simple Schottky-type configuration, we attempted to demonstrate the scalability of our method for fabricating a semitransparent solar cell. We used CVD-grown WS_2_ instead of mechanically exfoliated WSe_2_ and WS_2_ to fabricate the large-scale TMD sample. The CVD-grown few-layered WS_2_ was then transferred to the substrate used for device fabrication by the polymer capping method (see Fig. 4a and the Methods section for more details). Figure 4b shows a typical optical microscope image of the CVD-grown WS_2_ used in this study. Multiple devices were fabricated over the entire centimeter-scale substrate region (Fig. 4c). Each device was composed of Ni and Pt asymmetric electrode pairs formed by conventional photolithography (Fig. 4d). Optical microscopy and PL mapping images revealed that several WS_2_ crystals were in contact with each other between each Ni–Pt electrode pair (Fig. 4e,f). Clear photogenerated features were obtained, as shown in Fig. 4h, indicating that the wafer-scale fabrication of a Schottky-type solar cell with few-layered TMD is possible with our method. The concrete value of PCE could not be estimated due to the uncertainty of the area of CVD-grown WS_2_ bridging between the two electrodes. However, judging from the *I*_sc_ and *V*_oc_ values, the PCE of the CVD-grown WS_2_ was lower than that of the exfoliated WS_2_, whereas the basic photovoltaic features of the CVD-grown WS_2_ were similar to those of the mechanically exfoliated WS_2_, indicating that the improvement in the material quality of CVD-grown WS_2_ should be important (Figs S9 and S10). Similar device structures could also be fabricated on a flexible and transparent Polyethylene naphthalate (PEN) substrate (Fig. 4g). Clear *V*_oc_ and *I*_sc_ values were observed even for the Schottky solar cell with few-layered WS_2_ on a PEN substrate (Fig. 4i). The relatively low fill factor may be due to the uncontrolled Fermi level without *V*_gs_, which can be solved by chemical doping to WS_2_. Even after device fabrication, the transparency (∼75% on average) was maintained (Fig. 4j). Figure 4k shows the potential of the TMD-based semitransparent solar cell in comparison to other types of semitransparent solar cells^1,2,4,5,31–35^. As demonstrated in Fig. 3, the PCE of the Schottky-type solar cell with few-layered TMD increased up to at least 0.7% (this data was measured on a SiO_2_ substrate), whereas an average transparency of more than 75% in the visible light range was maintained because of the atomically thin structures. The transparency could be further improved by using other transparent electrode materials such as ITO, graphene, and carbon nanotubes. The use of nanomaterials as electrodes could also contribute to minimizing the drawback of Schottky-type solar cell, where the effective area for power generation is limited due to the lateral device configuration. Because the original transparency of TMD (>90%) is much higher than that of other devices, TMD-based semitransparent solar cells are promising, especially for solar cell applications requiring very high transparency.

### Power generation mechanism

**Figure 5:**
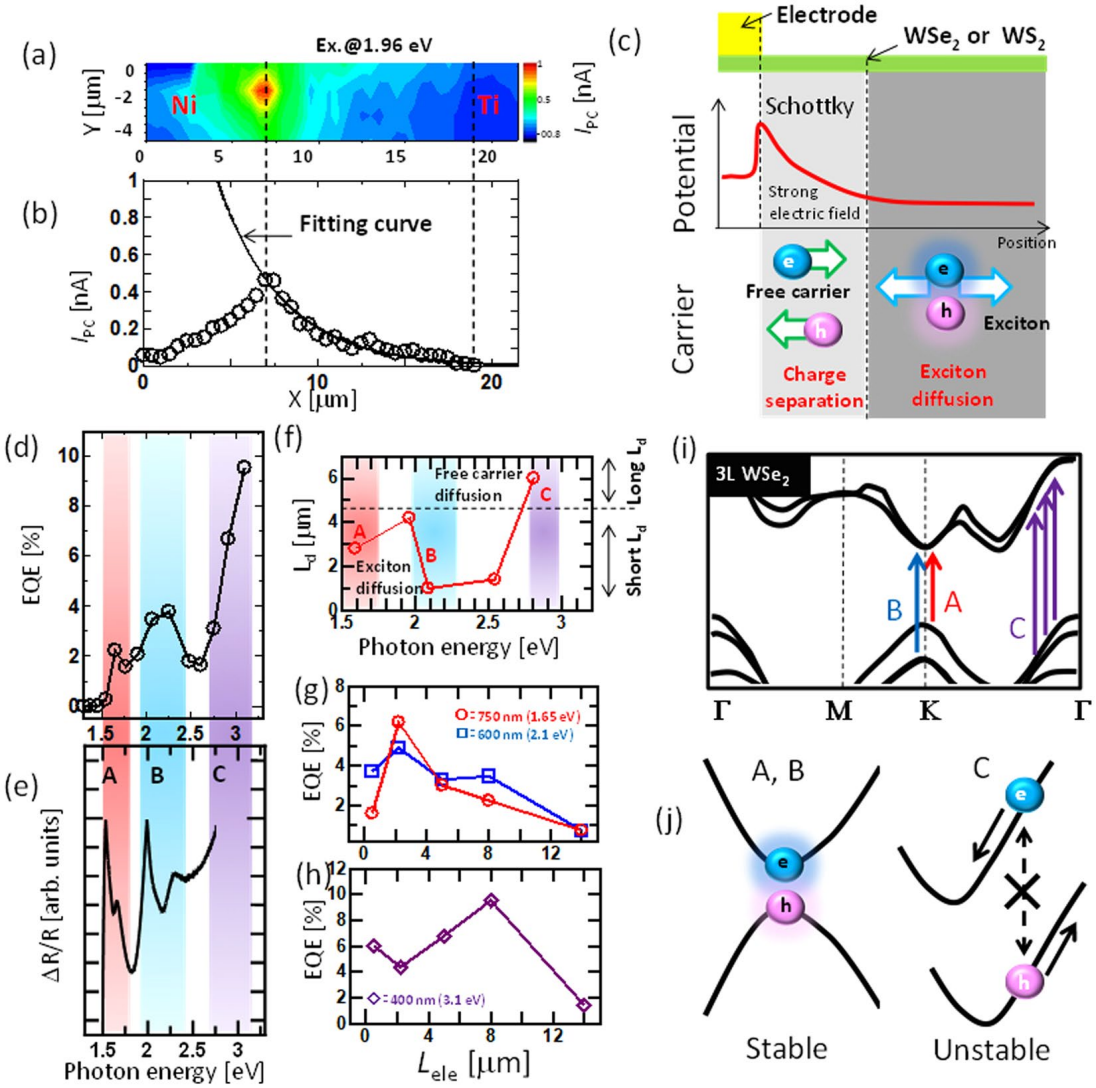
Power generation dynamics for few-layered WSe_2_ solar cell. (**a**,**b**) Typical (**a**) photocurrent mapping and (**b**) photocurrent line scan of few-layered WSe_2_ with an asymmetric electrode (Ti-Ni). (**c**) Schematic illustration of the photovoltaic power generation dynamics. (**d**,**e**) (**d**) EQE and (**e**) ΔR/R as a function of the incident photon energy. (**f**) L_d_ as a function of the incident photon energy. (**g**,**h**) EQE as a function of *L*_ele_ for different incident photon energies, (**f**) 1.65, 2.1 eV and (**g**) 3.1 eV. (**i**,**j**) (**i**) Schematic illustration of band structure of three-layered WSe_2_ (traced from ref.^37^) and (**j**) the exciton transition model for A,B (left), and C (right) excitons.

To further improve the PCE, it is important to understand the detailed mechanism of power generation with few-layered TMD in the Schottky-type configuration. We attempted to understand the detailed carrier dynamics for photovoltaic power generation with an asymmetric electrode. A line scan of the photocurrent mapping can give important information on carrier dynamics related to photogeneration^28–30^. Figure 5a and b show the photocurrent mapping and its line scan for an asymmetric electrode (Ni–Ti). The line scan profile of the photocurrent can be fitted with the following equation^29^.

1 $${I}_{PC}={I}_{S}\,\exp ({X}_{S}-X/{L}_{d})+{I}_{D}\,\exp (X-{X}_{D}/{L}_{d}),$$

where *I*_*S*_, *I*_*D*_, *X*_*S*_, *X*_*D*_ *X*, and *L*_*d*_ are the adjustable current amplitude for the source and drain regions, the contact positions for the source and drain regions, the distance from the source electrode, and the critical length for the power generation area, respectively. Because the photocurrent generation in WSe_2_ occurs only near the source (Ni) electrode, Equation (1) can be simplified as follows:

2 $${I}_{PC}={I}_{S}\,\exp ({X}_{S}-X/{L}_{d})$$

The line scan profile of the photocurrent mapping can be well fitted by Equation (2), leading to a value of *L*_*d*_ = 4.1 μm (Fig. 5a,b). The photocurrent generation in the Schottky-type TMD can be modeled with the following steps: (i) excitons are generated by incident photons and diffuse; (ii) excitons reach the depletion region at the Schottky barrier and can be dissociated into electrons and holes because of the strong electric field; and (iii) the dissociated carriers reach the electrode (=photocurrent) (Fig. 5c). Because processes (i) and (ii) determine *L*_d_, systematic investigations were carried out to identify the contribution of each process to *L*_d_. First, the potential profile was measured near the electrode with scanning Kelvin prove microscopy (SKPM) to identify the effect of process (ii). Through the line scan profile from SKPM, the length of the depletion layer at the contact region was found to be apploximately 217 nm, which is much shorter than *L*_*d*_(∼4.1 μm) (Fig. S11). This indicates that the effect of process (ii) is not very significant in determining *L*_*d*_, at least in our device. Then, the effect of process (i), exciton diffusion, was investigated. The EQE for the asymmetric electrode device (Ni–Ti) was measured as a function of excitation wavelength, and three clear peaks appeared at approximately 1.6, 2, and 3 eV (Fig. 5d). Three similar peaks also appeared in the PCE (Fig. S12). The peak positions agree well with the A, B, and C excitons in the differential reflectance (ΔR/R) spectra (Fig. 5e)^36^. This indicates that the photocurrent generation in the WSe_2_ Schottky device was mainly due to the excitonic response. The WS_2_ device (Fig. S13) showed a similar tendency. To identify the contribution of each exciton to solar power generation, *L*_d_ values obtained with different incident laser energies were measured with the same WSe_2_ device. Interestingly, the *L*_d_ value varied with the excitation energy: *L*_d_ = 3, 4, and 6 μm for 1.6 (A), 1.96 (B), and 2.8 eV (C) excitation (Fig. 5f). A similar trend was also found in the plot of EQE vs. *L*_ele_. For the 1.65 and 2.1 eV excitations (corresponding to A and B exciton excitations, respectively), the EQE decreased with decreasing *L*_ele_ from 2 to 14 μm (Fig. 5g), whereas the peak of EQE was approximately 8 μm with 3.1 eV excitation, corresponding to C excitation (Fig. 5h). The monotonic depletion of EQE vs. *L*_ele_ is known to be due to the loss of exciton collection in the depletion region, where the electrode distance is much longer than the exciton diffusion length^37^. The diffusion length of A excitons in TMD has been reported as 1–2 μm^38^, which agrees well with the *L*_d_ values for A and B excitons, indicating that *L*_d_ for A and B is mainly determined by the exciton diffusion length. The highest EQE for A and B excitons was obtained with *L*_ele_ = 2 μm, which is also consistent with the results of PCE vs. *L*_ele_ shown in Fig. 3b. In contrast to A and B excitons, relatively longer *L*_d_ values were observed for C exciton excitation, which was also observed for WS_2_ (Fig. S14). This can be explained as follows. Although the origin of C exciton generation is still unclear, it has been shown that the excitation at the band nesting region can easily dissociate electron and hole pairs due to the opposite band structures (Fig. 5i)^36,39^. According to this model, the excitons excited at approximately 3 eV may easily dissociate immediately after excitation before diffusion, and then the electrons and holes generated by incident photons can freely diffuse to the depression region near the Schottky contact, resulting in relatively longer *L*_d_. Similar features have also been reported for other organic solar cell devices^37^. These findings are very important in the design of device architectures for fully utilizing the potential ability of TMDs in semitransparent and flexible solar cell applications. Note that we did not take into account the indirect transition in 3 L WSe_2_ and WS_2_ to discuss the A, B, and C exciton dynamics for simplification. The first peak position of the EQE (PCE) spectra obtained with 3 L WSe_2_ and WS_2_ agreed well with the PL peak of the A exciton (Fig. S13d), and the A exciton peak energy in PL was almost the same for 1 L, 2 L, and 3 L WSe_2_ and WS_2_ (Fig. S13d). Thus, the explanation with a direct transition at the K point in 3 L WSe_2_ and WS_2_ should be reasonable (Fig. 5i,j). It should be mentioned that the dynamics for photo generated carriers are also sensitive to the band bending near the contact region, which will be discussed in a future work.

## Conclusions

We developed a high-performance Schottky-type solar cell with few-layered WSe_2_ and WS_2_. The Pd–Ni (Ni–Pt) electrode showed the highest PCE because of the asymmetric contact of Pd–WSe_2_ (Ni–WS_2_) and Ni–WSe_2_ (Pt–WS_2_) as ohmic-like and Schottky-type contacts, respectively. Based on the systematic investigation of the electrode type and structure adjustment, the PCE was improved by a factor of approximately 3000 compared with that before the adjustment (symmetric electrode), reaching a value of 0.7%, which is the highest value among solar cells with similar TMD thicknesses. The scalability of our method was also proven by forming a large-scale semitransparent solar cell on a PEN substrate with few-layered WS_2_. The systematic investigation of photocurrent mapping and the wavelength dependence of PCE and EQE revealed that the power generation dynamics differed depending on the exciton type. The effective power generation length for C exciton excitation was longer than those for A and B excitons, which can be explained by the band nesting effects of C excitons. These findings for a Schottky-type solar cell with few-layered TMD should contribute to the development of practical solar cell devices based on TMDs with high transparency and flexibility.

## Methods

### Structural characterization of TMD

The structures of TMD were characterized by optical microscopy, Raman and PL spectroscopy, and spatial mapping measurements with a 632.8-nm He-Ne laser and a 488-nm Ar laser, differential reflectance spectroscopy with a halogen lamp as a light source, AFM, and SEM.

### Device fabrication on the small size SiO_2_/Si substrate

First, few-layered TMD was prepared by mechanical exfoliation from a bulk crystal (2D semiconductor) with blue tape (HAKUTO) and transferred to a SiO_2_ (300 nm)/Si substrate. Then, conventional photolithography, electron beam lithography, vacuum evaporation of metal, and lift off were used to fabricate the symmetric and asymmetric devices. For the suspended device fabrication, TMD was position-selectively transferred to the electrode pattern by a homemade transfer system with a micro positioner and microscope.

### Device fabrication on the large size PEN substrate

The WS_2_ was grown on a SiO_2_ substrate by a conventional CVD method (Fig. 4a(i)). Then, the CVD grown WS_2_ was covered by a water-soluble polymer (Fig. 4a(ii) and transferred to the asymmetric electrodes (Fig. 4a(iii, vii)), which was separately prepared on a large size PEN substrate (Fig. 4a(iv-vi). The polymer was carefully removed by soaking in a water, obtaining the suspended WS_2_ device between asymmetric electrodes in a large size transparent and flexible substrate (Fig. 4a(viii)).

### Work function analysis

The work functions of metals were measured by photoelectron yield spectroscopy. A thick metal film (∼10 nm) was prepared by thermal evaporation on a SiO_2_/Si substrate, which was the same used for device fabrication.

### Electrical measurements

Device performance was measured with a vacuum probe station with a semiconductor parameter analyzer (HP 4155 C) at room temperature.

### Solar cell performance measurement

The I–V measurements were performed with a semiconductor parameter analyzer (HP 4155 C) and an Asahi spectra HAL-320 solar simulator (300 W Xenon ramp with an AM1.5 filter). The I–V curves for the estimation of accurate PCE were measured with a reverse to forward bias (−1 to 1 V), 10 mV step size and 20 ms dwell time and no difference in I-V curves was observed between forward and reverse sweep direction. The power of solar simulator (100 mW/cm^2^) was calibrated with an AIST-certificated standard solar cell (AK-100, KONICA MINOLTA JAPAN, INC.). The spectra mismatch was also considered for the calibration. No difference in power conversion efficiency was observed with and without masks (aperture size: 0.19 cm^2^). We used the total area (measured by SEM and OM) of suspended TMD between two electrodes as an active area for the estimation of PCE. The EQE spectrum was measured under continuous illumination by monochromatic light obtained using an arc lamp and a monochromator. The calibrated photodetector (Newport 818-UV) and thrermopile sensor (Newport 919P-003-10) were used for accurate lamp power measurement.

### Photocurrent mapping measurements

The line scan and mapping measurements of the photocurrent were performed with a homemade current probe system with Raman and PL mapping measurements. A multiple-wavelength laser was used to tune the excitation energy (442 nm He-Cd, 488 nm Ar, 607 nm, semiconductor, 632.8 nm He-Ne, 780 nm semiconductor laser), where the laser spot size was ∼1 μm (x100 objective).

## Electronic supplementary material

Dataset
