# Supplementary material for: Schottky solar cell using few-layered transition metal dichalcogenides toward large-scale fabrication of semitransparent and flexible power generator
Source: Sci Rep. 2017 Sep 20;7:11967. doi: 10.1038/s41598-017-12287-6 (PMC5607301; doi:10.1038/s41598-017-12287-6)
Supplement: Supplementary file 1 — Dataset [file 41598_2017_12287_MOESM1_ESM.doc]

**Supplemental data set**

Schottky solar cell using few-layered transition metal dichalcogenides toward large-scale fabrication of semitransparent and flexible power generator

Toshiki Akama^Ϯ^, Wakana Okita^Ϯ^, Reito Nagai, Chao Li, Toshiro Kaneko, and Toshiaki Kato*

**Table S1|** Type and basic features of solar cells with TMD.

[S1] Groenendijk, D.J. *et al*. Photovoltaic and photothermoelectric effect in a double-gated WSe_2_ device. *Nano Lett.* **14**, 5846-5852 (2014).

[S2] Pospischil, A., Furchi, M. M. & Mueller, T. Solar-energy conversion and light emission in an atomic monolayer p–n diode. *Nat. Nanotechnol.* **9**, 257-261 (2014).

[S3] Baugher, B. W. H., Churchill, H. O. H., Yang, Y. & Jarillo-Herrero, P. Optoelectronic devices based on electrically tunable p–n diodes in a monolayer dichalcogenide. *Nat. Nanotechnol.* **9**, 262-267 (2014).

[S4] Memaran, S. *et al*. Pronounced photovoltaic response from multilayered transition-metal dichalcogenides pn-junctions. *Nano Lett.* **15**, 7532-7538 (2015).

[S5] Wi, S. *et al*. Enhancement of photovoltaic response in multilayer MoS_2_ induced by plasma doping *ACS Nano* **8**, 5270-5281 (2014).

[S6] Deng, Y. *et al*. Black phosphorus-monolayer MoS_2_ van der Waals heterojunction p-n diode. *ACS Nano* **8**, 8292-8299 (2014).

[S7] Cheng, R. *et al*. Electroluminescence and photocurrent generation from atomically sharp WSe_2_/MoS_2_ heterojunction p−n diodes. *Nano Lett.* **14**, 5590-5597 (2014).

[S8] Lee, C.-H. *et al*. Atomically thin p–n junctions with van der Waals heterointerfaces. *Nat. Nanotechnol.* **9**, 676-681 (2014).

[S9] Gong, Y. *et al*. Two-step growth of two-dimensional WSe_2_/MoSe_2_ heterostructures. *Nano Lett.* **15**, 6135-6141 (2015).

[S10] Fontana, M. *et al*. Electron-hole transport and photovoltaic effect in gated MoS_2_ Schottky junctions. *Sci. Rep.* **3**, 1634-1-5 (2013).

.


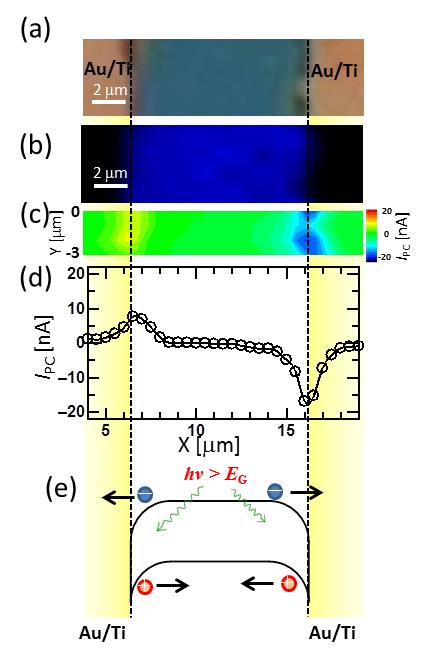


**Figure S1| Photocurrent mapping with symmetric electrode without *V*_ds_.** (a) Optical microscopy, (b) Raman intensity mapping, (c) photocurrent mapping, (d) photocurrent line scan, and (e) band diagram of few-layered WSe_2_ with symmetric electrodes (Ti-Ti)


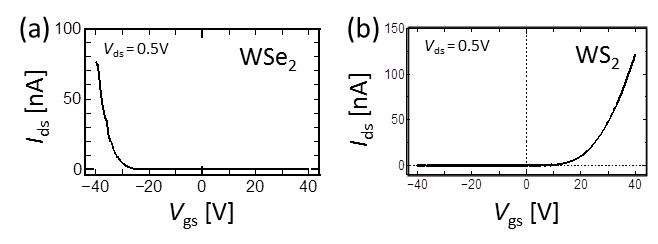


**Figure S2| Basic electrical features of WSe_2_ and WS_2_ device.** (a,b) Typical *I*_ds_-*V*_gs_ curves of (a) WSe_2_ and (b) WS_2_ devices used in this study.

**Figure S3| Electrical band alignment of monolayer (a) WSe_2_ and (b) WS_2_. Electrical band of WSe_2_ and WS_2_ is drawn by using the value shown in ref. [S11].**

[S11] F. A. Rasmussen and K. S. Thygesen, Computational 2D Materials Database: Electronic Structure of Transition-Metal Dichalcogenides and Oxides, J. Phys. Chem. C 119, (2015) 13169−13183.


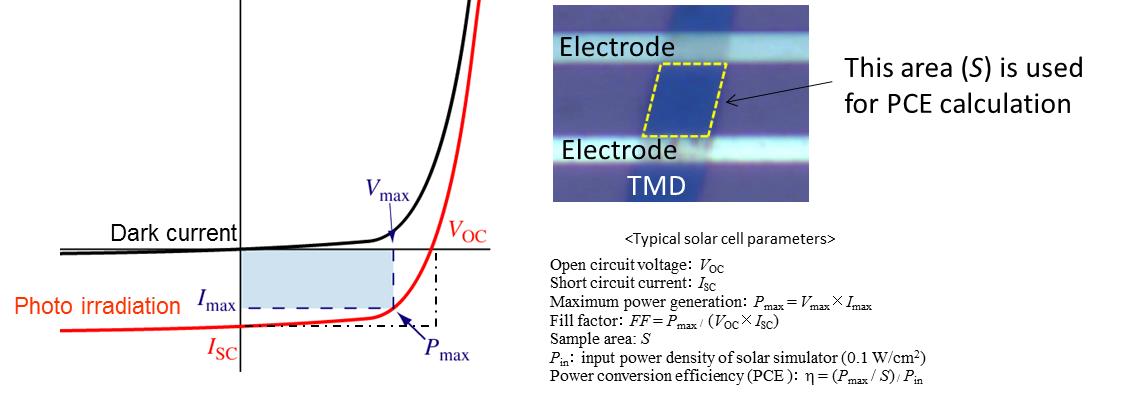


**Figure S4| Calculation method of typical solar cell parameters.**


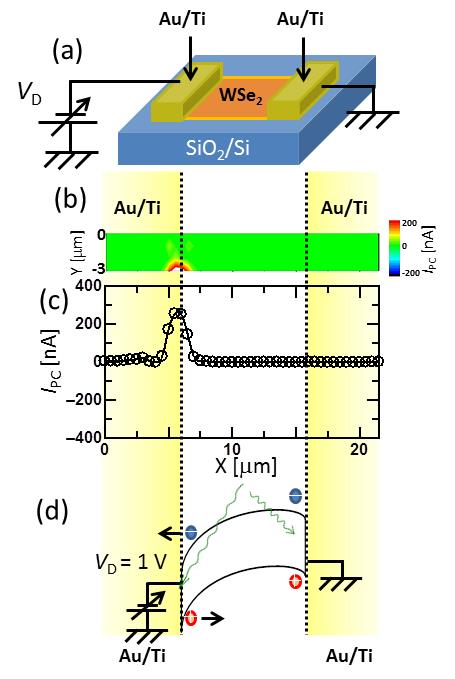


**Figure S5| Photocurrent mapping with the symmetric electrode with *V*_ds_ =1 V.** (a) Device configuration, (b) photocurrent mapping, (c) photocurrent line scan, and (d) band diagram of few-layered WSe_2_ with symmetric electrodes (Ti-Ti).


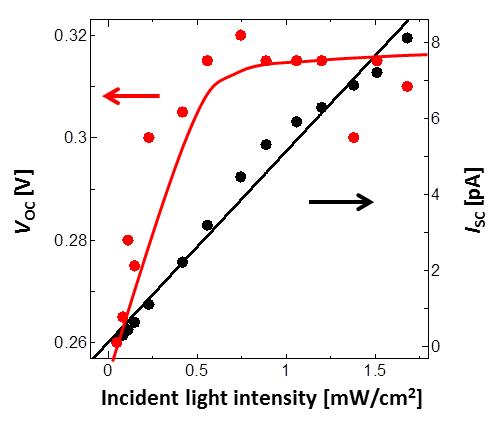


**Figure S6| Dependency of solar cell features for few-layered WS_2_ on the incident light intensity.** Typical *I*_sc_ (red) and *V*_oc_ (black) of few-layered WS_2_ with asymmetric electrode pairs (Pt-Ni) under different incident light (600 nm) intensities.


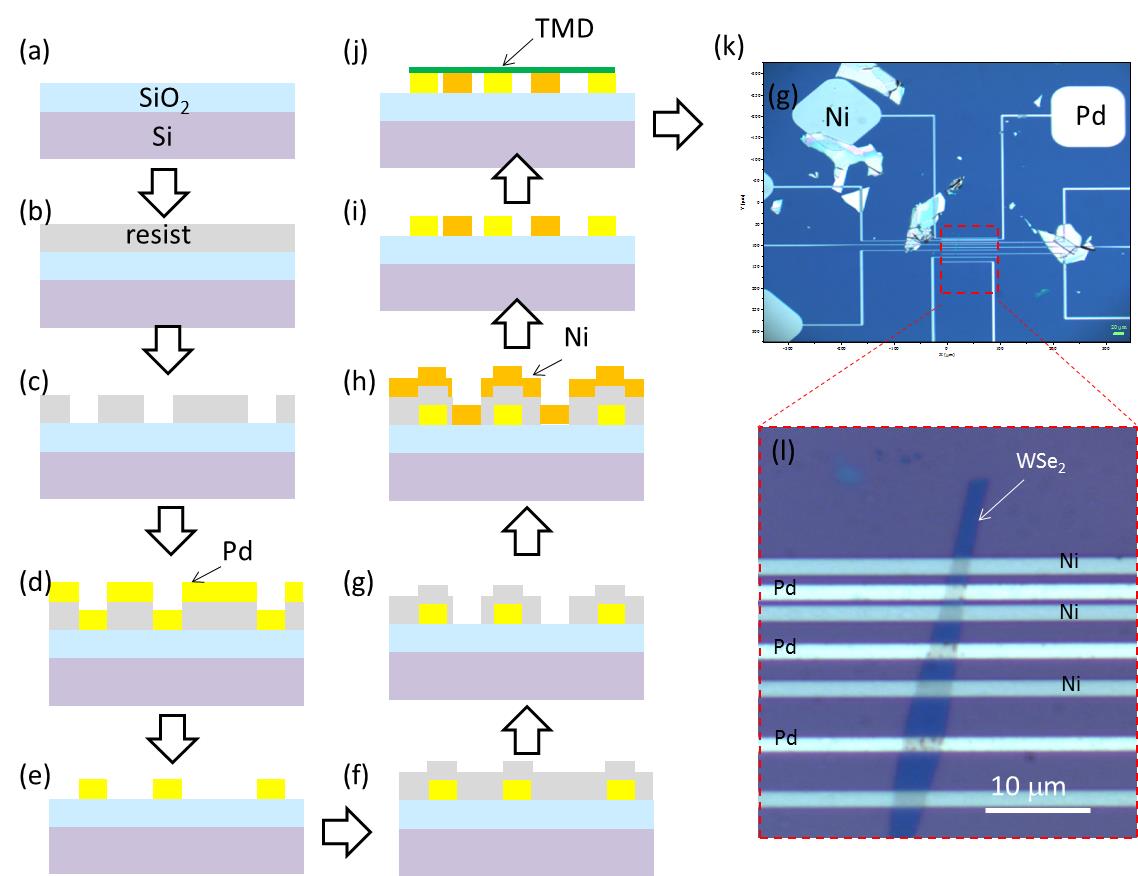


**Figure S7| Typical fabrication process of suspended solar cell device.** (a-j) Step by step model of fabrication process relating with electron beam lithography and lift off process. (k,j) Typical (k) low and (l) high magnification OM image of suspended WSe_2_ device with Pd-Ni electrode.


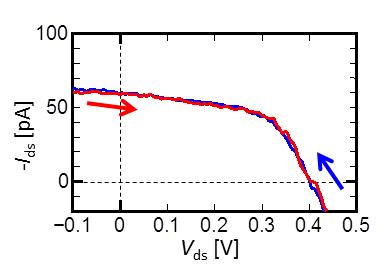


**Figure S8| Stability of solar cell performance.** Typical *I*_ds_ – *V*_ds_ curve of few-layered WSe_2_ with asymmetric electrode pairs (Pd-Ni) measured with forward (red) and reverse (blue) sweep direction under solar simulator irradiation.


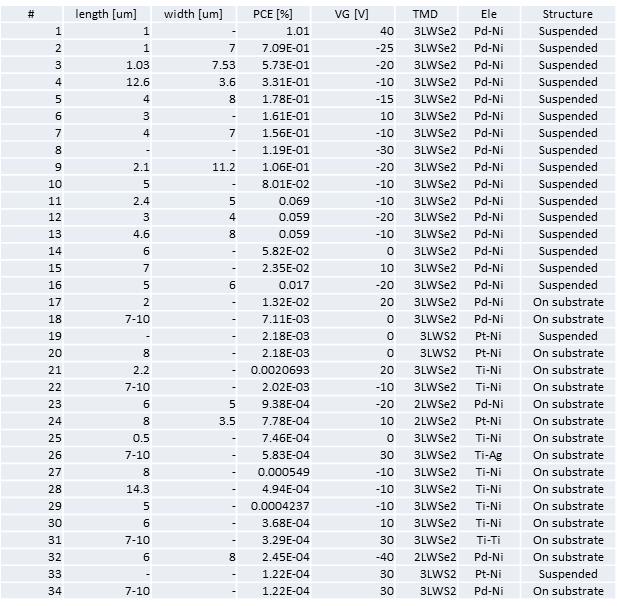


**Table S2| Detailed data list of device used in this study.**


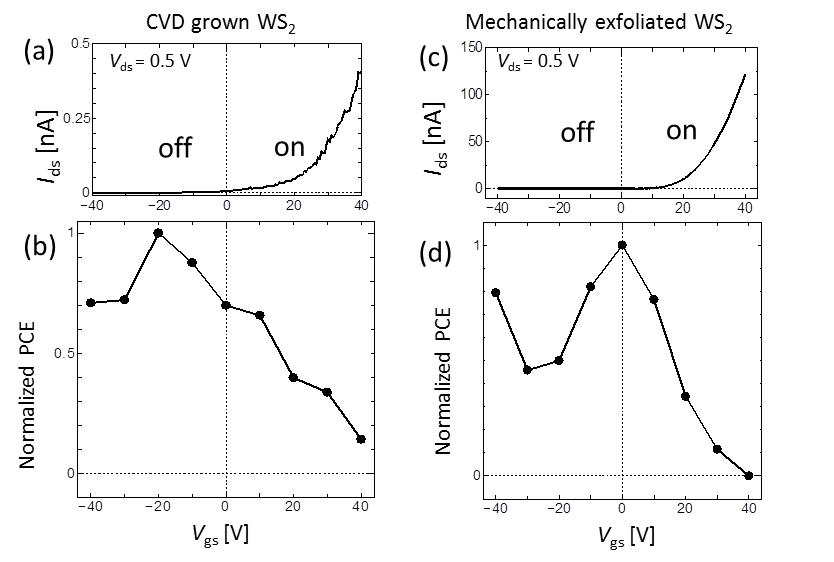


**Figure S9|** **Comparison of CVD grown WS_2_ and exfoliated WS_2_.** Typical (a,c) I_ds_-V_gs_ curves and (b,d) normalized PCE as a function of *V*_gs_ for (a,b) CVD-grown and (c,d) mechanically exfoliated WS_2_.


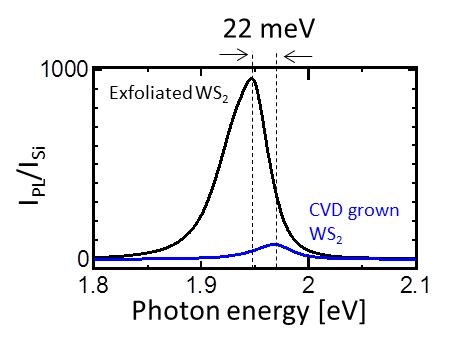


**Figure S10| Typical PL spectra of exfoliated monolayer WS_2_ and CVD grown monolayer WS_2_.** PL intensity (I_PL_) was normalized by Raman peak intensity of Si (I_Si_) comes from substrate. I_PL_/Isi of exfoliated WS_2_ is much higher than that of CVD grown WS_2_. Peak energy of PL spectrum of CVD grown WS_2_ is 22 meV up shifted compared with that of exfoliated WS_2_. These indicate that the defects and impurities may be introduced to CVD grown WS_2_.


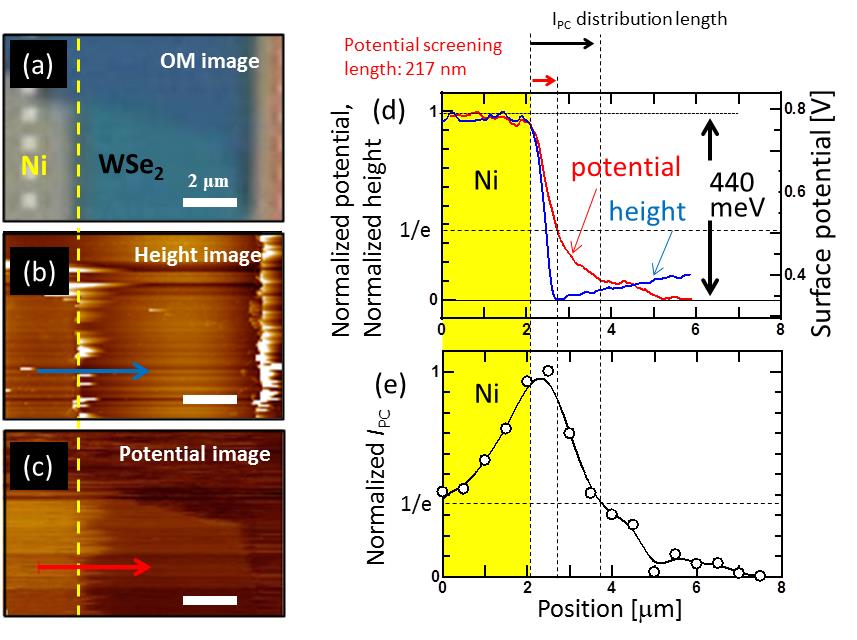


**Figure S11| Estimation of depression length at the contact region.** (a) Optical microscope image, (b) height image obtained with AFM, and (c) potential image measured by SKPF of WSe_2_ in partial contact with Ni. (d,e) Line profile of (d) the normalized height (blue) and the potential (red) and (e) the photocurrent (*I*_pc_) from Ni to the WSe_2_ region. Absorute value of potential difference between Ni and WSe_2_ is about 440 meV, which is reasonable value as the Schottky barrier height estimated from the band diagram shown in Fig. 2d.


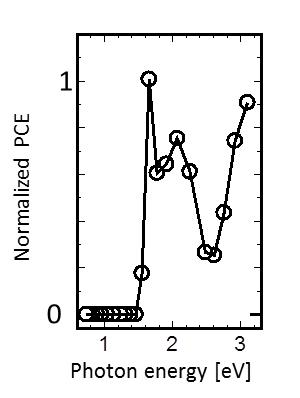


**Figure S12| PCE vs. photon energy.** PCE as a function of the incident photon energy with WSe_2_.


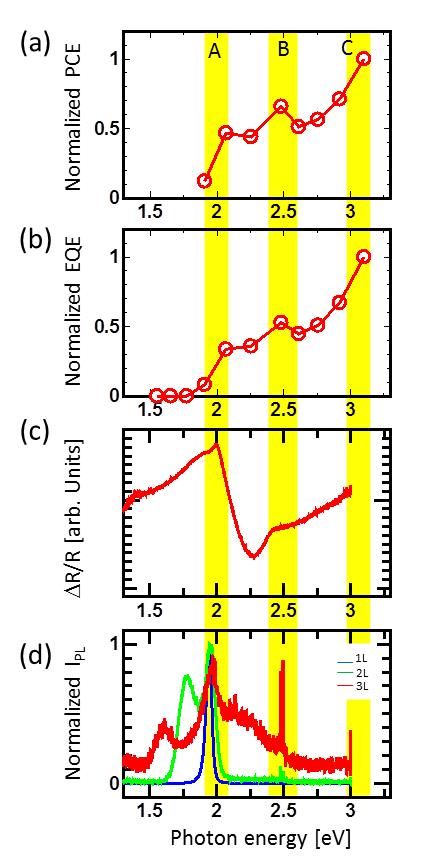


**Figure S13| Dependence of incident photon energy with WS_2_.** Dependence of the incident photon energy on (a) PCE, (b) EQE, and (c) ΔR/R for the tri-layered WS_2_ device. (d) Typical PL spectra of 1L (blue), 2L (green), and 3L (red) WS_2_.


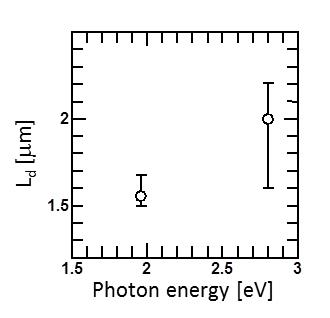


**Figure S14| L_d_ vs. photon energy.** Plot of L_d_ as a function of the incident photon energy for WS_2_ with the asymmetric electrode pair.
